# Decoding the Dual Defense: Mechanistic Insights into Antioxidant and Anti-Inflammatory Phytochemicals

**DOI:** 10.3390/plants14223531

**Published:** 2025-11-19

**Authors:** Seok-Geun Lee

**Affiliations:** 1 Department of Biomedical Science and Technology, Kyung Hee University, Seoul 02447, Republic of Korea; seokgeun@khu.ac.kr; Tel.: +82-2-961-2355; 2 BioNanocomposite Research Center, Kyung Hee University, Seoul 02447, Republic of Korea

## 1. Introduction

Chronic diseases such as diabetes, cardiovascular disease, neurodegeneration, and cancer share a common pathogenic thread: they are driven by excessive oxidative stress and unchecked inflammatory responses [1]. Oxidative stress and inflammation are tightly interconnected processes. Reactive oxygen species (ROS) can trigger inflammatory signaling, and inflammation in turn amplifies oxidative damage, creating a detrimental positive feedback loop [1]. Natural products continue to provide structurally diverse molecules that modulate these pathways, and notable examples include acetylsalicylic acid and paclitaxel, both of which are derived from plants [2,3]. Yet, countless bioactive phytochemicals remain undiscovered, and the escalating global burden of chronic disease necessitates continuous innovation in therapeutic strategies. This Special Issue, “Antioxidant and Anti-inflammatory Activities of Plant Extracts and Phytochemicals”, provides a platform for research on plant-derived compounds capable of modulating oxidative stress and inflammatory processes. The contributions illustrate a shift beyond simple phytochemical quantification toward detailed mechanistic analyses. By employing advanced phytochemical profiling and cellular assays, these studies reveal how plant extracts and phytochemicals influence fundamental pathways underlying oxidative stress and chronic inflammation. Here, we summarize the contribution’s findings and highlight emerging themes.

## 2. The Foundation: Deepened Phytochemical Profiling

Modern phytochemical research demands precise identification of bioactive molecules. Articles in this Special Issue showcase the extraordinary chemical diversity underpinning plant efficacy. For example, extracts from the ethnomedicinal vine *Fissistigma oldhamii* were found to be rich in alkaloids, terpenoids, and flavonoids [4]. A homeopathic mother tincture of *Viscum album* (European mistletoe) was analyzed via Liquid Chromatography–Mass Spectrometry (LC–MS) and High-Performance Liquid Chromatography (HPLC), revealing a complex metabolite profile and viscotoxin content comparable to that of commercial products. This tincture exhibited cytotoxic and antioxidant activities while remaining safe in vivo (LD_50_ > 2 g/kg) [5]. Gas Chromatography–Mass Spectrometry (GC–MS) analysis of *Sesamum prostratum* Retz. identified 32 compounds, and notably, the ethanol, ethyl acetate, and acetone fractions showed distinct phytochemical profiles and demonstrated free-radical scavenging activity in 2,2-diphenyl-1-picrylhydrazyl (DPPH), 2,2′-azino-bis(3-ethylbenzothiazoline-6-sulfonic acid) (ABTS), and hydrogen peroxide (H_2_O_2_) assays [6]. Three glycosides isolated from *Ternstroemia lineata* flower buds and fruits protected yeast cells from H_2_O_2_-induced stress, further highlighting the presence of antioxidant constituents [7]. In another study, a GC-Flame ionization detector (GC-FID) was used to compare lyophilized versus oven-dried extracts of *Manilkara zapota* (sapodilla) pulp, peel, and seeds [8]. While water content remained unchanged post-harvest, the drying method influenced dietary fiber and carbohydrate composition. GC-FID revealed changes in fatty acid profiles, although the dried extracts showed only weak antibacterial (minimum inhibitory concentration > 1000 µg/mL) and antioxidant (IC_50_ > 2000 µg/mL) activities [8].

An important next step is linking phytochemical profiles to bioactivity. For instance, the anticancer activity of *Pittosporum angustifolium* is attributed to phenolic metabolites identified through rigorous LC-MS profiling—most notably chlorogenic acid and rosmarinic acid [9]. Likewise, species of the *Hypericum* genus were found to contain high levels of potent radical scavengers such as rutin and quercetin [10]. Such detailed molecular mapping, often employing advanced techniques like ultra-HPLC (UPLC) coupled with MS, is paramount for effective bioassay-guided fractionation and for validating traditional medicinal uses of plants [5,6,7,8,9,10,11,12].

## 3. Anti-Inflammatory Mechanisms: Targeting Key Regulatory Hubs

A significant body of work focuses on how plant derivatives inhibit the core cellular machinery of inflammation, primarily the Nuclear Factor-κB (NF-κB) and Mitogen-Activated Protein Kinase (MAPK) pathways [13,14]. For example, cannabichromene isolated from hemp demonstrates potent anti-inflammatory activity by suppressing nitric oxide (NO) production and downregulating the expression of inducible nitric oxide synthase (iNOS), interleukin (IL)-1β, IL-6, and tumor necrosis factor (TNF)-α in lipopolysaccharide (LPS)-activated macrophages [13]. It also blocks NF-κB and MAPK signaling and alleviates inflammation in a carrageenan-induced mouse paw edema model [13]. Similarly, compounds from *F. oldhamii* exhibit anti-inflammatory effects. Isocorydine prevents the nuclear translocation of NF-κB p65, thereby reducing pro-inflammatory cytokine release, and the propenamide derivative Z23 downregulates cyclooxygenase (COX)-2 and iNOS gene expression to resolve inflammation [4].

Crude plant extracts and their enriched fractions often act on multiple inflammatory targets simultaneously. An illustrative example is an aqueous extract of *Rhus trilobata* and its active fraction F6, which significantly reduced mRNA levels of IL-1β, IL-6, TNF-α, and COX-2 in LPS-stimulated macrophages, leading to lower prostaglandin E2 (PGE2) production [12]. In an LPS-induced rat paw edema model, the same fraction mitigated swelling and inflammatory cell infiltration. UPLC-MS/TOF (Time-of-Flight detection) analysis identified multiple putative anti-inflammatory compounds within this faction [12]. Such multi-target inhibition supports the therapeutic advantage often ascribed to complex phytochemical mixtures over single isolated molecules, underscoring the synergy inherent in many herbal remedies.

## 4. Antioxidant–Inflammation Crosstalk and Novel Pathways

Antioxidant and anti-inflammatory responses are intimately connected, and a major finding across this Special Issue is the regulatory interplay between the primary cellular antioxidant sensor Nuclear Factor Erythroid 2-Related Factor 2 (NRF2) and the master inflammatory regulator NF-κB. Many plant extracts display antioxidant potential in standard radical scavenging assays like DPPH, ABTS, and H_2_O_2_ [6,15], but the key advance lies in demonstrating how activating antioxidant pathways can concurrently dampen inflammation.

A detailed study on *Polygonum aviculare* L. (PAL) exemplifies this dual action [11]. The hydroethanolic extract of PAL activates the NRF2 pathway, leading to upregulated expression of the antioxidant enzyme Heme Oxygenase (HO)-1. This HO-1 induction proved to be a prerequisite for PAL’s anti-inflammatory effect. Inhibiting HO-1 abolished PAL’s suppression of NF-κB-mediated iNOS expression and the resulting NO production [11]. By inducing HO-1 via NRF2, the extract concurrently inhibited COX-2 expression and reduced PGE2 levels. These effect were largely attributed to PAL’s flavonoid constituents, especially kaempferol and quercetin [11]. Similarly, *Bauhinia forficata* Link extract protects human keratinocytes from H_2_O_2_-induced oxidative damage by activating the NRF2/PTEN-Induced Kinase (PINK)1 pathway, thereby enhancing antioxidant defenses and mitochondrial quality control, while simultaneously suppressing NF-κB signaling to lower the release of pro-inflammatory cytokines [14]. These examples underscore a reciprocal relationship between NRF2-driven antioxidant responses and NF-κB-driven inflammatory responses as a central mechanism of phytochemical action.

The concept of concurrently suppressing inflammation and oxidative stress extends into cancer chemoprevention and other chronic disease contexts. For instance, a chalcone compound, 2′,4′-dihydroxy-6′-methoxy-3′,5′-dimethylchalcone (DMC), isolated from *Cleistocalyx nervosum* seeds, exhibited chemopreventive effects in a rat model of hepatocarcinogenesis [16]. Oral administration of DMC (10 mg/kg), as well as the crude seed extract, significantly reduced the number and size of diethylnitrosamine-induced preneoplastic liver lesions, decreased aberrant cell proliferation, and promoted apoptosis in the lesions [16]. In another study, high-order oligomeric alkaloids from *Securinega virosa* (fluevirosines A–H and fluevirosinines A–J), which possess anti-inflammatory and anti-proliferative activities, were investigated via molecular docking [17]. Despite their large size, these norsecurinine-type alkaloids were predicted to bind within the α-tubulin drug-binding pocket, and the analysis highlighted specific trimers, tetramers, and pentamers as promising tubulin-binding ligands with potential anticancer activity [17].

An exploration of *P. angustifolium* and *Terminalia ferdinandiana* further emphasizes the importance of synergistic multi-component efficacy [9]. LC-MS/MS and GC-MS analyses of these plants’ extracts identified chlorogenic acid, neochlorogenic acid, bergapten, berberine, and rosmarinic acid as major constituents. However, the anticancer and antibacterial activities markedly decreased after fractionation, implying that the synergy among the original constituents was critical for full bioactivity [9]. These findings caution that separating phytochemicals can sometimes diminish efficacy by disrupting natural synergistic interactions [9].

Finally, a comprehensive review of *F. oldhamii* (Annonaceae), a traditional Chinese medicinal plant used to treat rheumatic pain and inflammation, cataloged more than 70 secondary metabolites, including aporphine alkaloids, anthraquinones, terpenoids, and flavonoids identified in various plant parts [4]. Many of these constituents exhibit anti-inflammatory, antioxidant, anticancer, or antimicrobial effects and are known to modulate pathways such as NF-κB and cellular oxidative stress responses [4]. The review highlights that network pharmacology approaches will be needed to pinpoint the most active compounds and their precise molecular targets within such complex phytochemical mixtures [4].

## 5. Novel Antioxidant Strategies and Resource Optimization

Beyond well-established radical scavenging activity, this Special Issue reveals sophisticated and unexpected antioxidant mechanisms employed by plant compounds. A striking example is the mechanism proposed for certain *T. lineata* phytochemicals, such as hydroxytyrosol-1-O-glucoside. Molecular docking suggests that these compounds delay H_2_O_2_-induced oxidative stress in yeast cells by physically interacting with aquaporin (AQP) channels, including *Saccharomyces cerevisiae* AQP3 and human AQP7, and thereby regulate the transport of H_2_O_2_ into cells [7]. This mode of antioxidant action, which involves modulating H_2_O_2_ permeability rather than directly scavenging free radicals, offers new insights into how phytochemicals can protect cells from oxidative damage.

Detailed phytochemical analyses in this Issue also confirm a strong link between polyphenolic content and antioxidant capacity. Extracts from various *Hypericum* species, including under-studied species like *H. hirsutum*, *H. barbatum*, and *H. rochelii*, were found to have exceptionally high total phenolic and flavonoid contents, correlating with robust antioxidant effects in multiple assays [10]. Likewise, both ethanolic and aqueous extracts of hemp (*Cannabis sativa*) contain abundant polyphenols and exhibit strong scavenging activity against DPPH and hydroxyl (OH) radicals, reinforcing their potential value in combating oxidative stress-related conditions such as diabetes [15]. The *V. album* mother tincture mentioned above is another case in point. It was confirmed to harbor a rich array of phenolics, including chlorogenic acid and kaempferol, which contribute to its antioxidant activity [5].

Translating these phytochemical benefits into practical therapeutics requires innovation in formulation and resource use. One study optimized a multi-component herbal mixture (turmeric, coffee, and chili extracts in a 1:3:4 ratio) for maximal antioxidant and anti-inflammatory efficacy, then successfully encapsulated this combination into nanostructured lipid carriers to enhance its stability and bioavailability [18]. Such nanocarrier-based formulation significantly improved the delivery and therapeutic potential of the phytochemicals, validating the strategy of combination therapy and addressing known limitations in oral bioavailability [18].

Sustainable resource utilization is another prominent theme. For example, by-products of *Rosa damascena* (Damask rose) essential oil distillation were examined as inexpensive sources of antioxidants [19]. The distilled waste hydrosol and rose dreg fractions were found to contain the highest phenolic and flavonoid levels and exhibited strong antioxidant and anti-melanogenesis activity, although they showed no notable antimicrobial or anti-inflammatory effects [19]. This finding underscores that agricultural or industrial waste streams from aromatic and medicinal plants can be repurposed as value-added ingredients for functional foods, cosmetics, or therapeutic formulations. Leveraging such by-products not only maximizes resource efficiency but also aligns with sustainable development goals in natural product research.

## 6. Outlook

The future of natural products research will center on optimizing delivery systems and maximizing resource utilization for phytochemicals with dual antioxidant and anti-inflammatory activities. The successful encapsulation of polyphenol-rich plant combinations into nanostructured carriers demonstrates how advanced formulation techniques can dramatically enhance bioavailability and therapeutic effect [18]. Concurrently, the discovery of high phenolic content and potent antioxidant activity in by-products such as *R. damascena* hydrosols and residues highlights a promising avenue for developing sustainable, value-added therapeutics from botanical waste streams [19]. The investigation of lesser-known plant species also holds great promise. For instance, under-explored *Hypericum* species were shown to exceed the well-known *H. perforatum* in phenolic content and to exhibit not only strong antioxidant capacities but also enzyme-inhibitory and antibacterial activities (e.g., against methicillin-resistant *Staphylococcus aureus*) [10]. Such findings emphasize the value of bioprospecting native and under-investigated plants for novel bioactive compounds [9].

Collectively, the articles in this Special Issue reaffirm the integral role of plants in drug discovery, particularly in addressing pathologies driven by chronic oxidative stress and inflammation. By providing molecular-level insights—whether through elucidating the reciprocal regulation of NRF2 and NF-κB, the broad inhibition of pro-inflammatory mediators, or the discovery of novel antioxidant mechanisms such as AQP modulation—this body of work strengthens the scientific foundation for the medicinal use of plant extracts and phytochemicals. Future research should build on these mechanistic breakthroughs to accelerate the clinical translation of potent plant-derived antioxidant and anti-inflammatory agents.

## Data Availability

No new data were created or analyzed in this study. Data sharing is not applicable to this article.

## Abbreviations

The following abbreviations are used in this manuscript:

ROS	Reactive oxygen species
LC	Liquid chromatography
MS	Mass spectrometry
HPLC	High-performance LC
GC	Gas chromatography
DPPH	2,2-diphenyl-1-picrylhydrazyl
ABTS	2,2′-azino-bis(3-ethylbenzothiazoline-6-sulfonic acid
FID	Flame ionization detector
UPLC	Ultra-HPLC
NF-κB	Nuclear factor-κB
MAPK	Mitogen-activated protein kinase
NO	Nitric oxide
iNOS	Inducible NO synthase
IL	Interleukin
TNF	Tumor necrosis factor
LPS	Lipopolysaccharide
COX	Cyclooxygenase
PGE2	Prostaglandin E2
TOF	Time-of-flight detection
NRF2	Nuclear Factor Erythroid 2-Related Factor 2
HO-1	Heme oxygenase-1
PAL	*Polygonum aviculare* L.
PINK1	PTEN-induced kinase 1
DMC	2′,4′-dihydroxy-6′-methoxy-3′,5′-dimethylchalcone
AQP	Aquaporin
